# Different Pattern of Cardiovascular Impairment in Methylmalonic Acidaemia Subtypes

**DOI:** 10.3389/fped.2022.810495

**Published:** 2022-02-23

**Authors:** Ying Liu, Ling Yang, Ruixue Shuai, Suqiu Huang, Bingyao Zhang, Lianshu Han, Kun Sun, Yurong Wu

**Affiliations:** ^1^Department of Pediatric Cardiology, Xinhua Hospital, School of Medicine, Shanghai Jiao Tong University, Shanghai, China; ^2^Department of Pediatrics, Changzheng Hospital, Naval Medical University, Shanghai, China; ^3^Department of Pediatric Genetic Endocrinology, Xinhua Hospital, School of Medicine, Shanghai Jiao Tong University, Shanghai, China

**Keywords:** methylmalonic acidaemia, homocystinuria, cobalamin C type, cardiac dysfunction, congenital heart disease, pulmonary arterial hypertension

## Abstract

Methylmalonic acidaemia (MMA) has been reported to be associated with cardiovascular involvement, especially for the combined type with homocystinuria. We have screened 80 control subjects and 99 MMA patients (23 isolated type and 76 combined type) using electrocardiograph and echocardiography. 32 cases (34%) of ECG changes were found including sinus tachycardia (*n* = 11), prolonged QTc interval (*n* = 1), I-degree atrioventricular block (*n* = 1), left axis deviation (*n* = 5) and T wave change (*n* = 14). By echocardiography, 8 cases of congenital heart disease were found in 4 combined MMA patients (5.3%) including ventricular septal defect (*n* = 2), atrial septal defect (*n* = 3), patent ductus arteriosus (*n* = 1) and coronary artery-pulmonary artery fistula (n =2). Pulmonary hypertension (*n* = 2) and hypertrophic cardiomyopathy (*n* = 1) in combined subtype were also noted. Moreover, echocardiographic parameters were analyzed by multiple regression to clarify the influence of different subtypes on cardiac function. It was found that the left ventricular mass index (LVMI) was significantly reduced only in combined subtype [*R* = −3.0, 95%CI (−5.4, −0.5), *P* = 0.017]. For left ventricle, the mitral E' velocity was significantly reduced [isolated type: *R* = −1.8, 95%CI (−3.3, −0.4), *P* = 0.016; combined type: *R* = −2.5, 95%CI (−3.5, −1.5), *P* < 0.001], the global longitudinal strain (GLS) was the same [isolated type: *R* = −1.4, 95%CI (−2.3, −0.4), *P* = 0.007; Combined type: *R* = −1.1, 95%CI (−1.8, −0.4), *P* = 0.001], suggesting weakened left ventricular diastolic and systolic functions in both subtypes. For right ventricle, only in combined subtype, the tricuspid E' velocity was significantly reduced [*R* = −1.4, 95%CI (−2.6, −0.2), *P* = 0.021], and the tricuspid annular plane systolic excursion (TAPSE) was the same [*R* = −1.3, 95%CI (−2.3, −0.3), P=0.013], suggesting impaired right ventricular systolic and diastolic function. In conclusion, isolated and combined types showed different pattern of cardiac dysfunction, specifically the former only affected the left ventricle while the latter affected both ventricles. And it is necessary to perform echocardiographic screening and follow up in both MMA subtypes.

## Introduction

**Figure 1:**
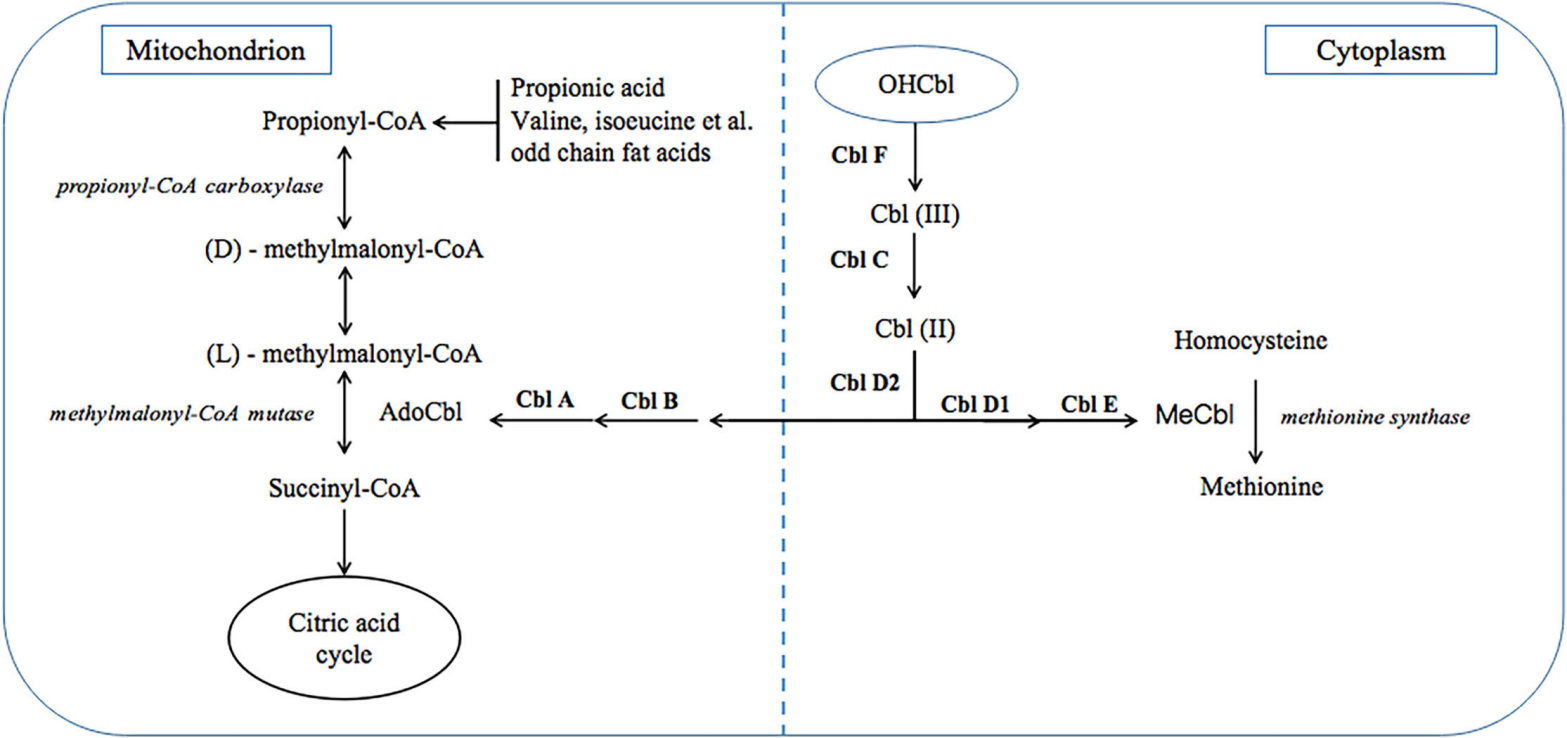
Metabolic pathway of cobalamin and MMA.

Methylmalonic acidaemia (MMA) is a class of diseases due to various inherited autosomal recessive gene defects, which result in impaired function of methylmalonyl-CoA mutase (MCM) or impaired intracellular cobalamin (Cbl) transport and processing (1, 2). Clinically, MMA can be divided into two types, isolated MMA and combined MMA which is also called MMA with homo-cystinuria (MMA/HCY). The former is due to MCM defects or Cbl A/B defects which only cause deficient adenosylcobalamin (AdoCbl) within mitochondrion, while other Cbl defects can also affect methylcobalamin (MeCbl) synthesis and results in the latter (Figure 1). In China, combined type is the most common type, and CblC defect accounts for most cases with related gene identified as *MMACHC* (3), which is located on chromosome 1p and responsible for CNCbl decyanase.

This disease has a broad spectrum of clinical manifestations. Most patients suffer from nervous system impairment and extranervous system involvement. Currently, cardiovascular involvement has begun to draw attention as an increasing number of cases have been reported in combined type, especially CblC patients. The first CblC patient with cor pulmonale as a complication was reported by Brandstetter et al. in 1990 (4). Profitlich et al. (5) conducted a retrospective study to analyse echocardiographic data in ten CblC patients and found that half of them had structural heart defects. In some patients, the sudden onset of pulmonary arterial hypertension (PAH) or renal hypertension can be a trigger of heart failure and progress into a life-threatening event (6–10).

Cardiomyopathy has been observed in many inherited metabolic diseases, such as Anderson-Fabry disease (11), as cardiac energetic impairment can play a causal role in cardiac dysfunction and vulnerability (12, 13). Both types of MMA patients have also shown manifestations such as dilated or hypertrophic cardiomyopathy.

Currently, no studies have investigated the cardiac function of MMA patients in the stable stage. Echocardiography has been a useful and non-invasive tool to measure cardiac structure, function and haemodynamic. Tissue Doppler imaging can provide an assessment of myocardial systolic and diastolic function in both ventricles (14), and a novel technique, speckle-tracking echocardiography (STE), can provide an accurate assessment of myocardial deformation and detect preclinical myocardial dysfunction when EF is normal (15–18).

Hence, we organized a screening of the cardiovascular system to assess cardiac function and clarify the incidence of congenital heart disease (CHD), cardiomyopathy and PAH in MMA patients and explore the difference between the subtypes.

## Methods and Materials

### Population

From April 2019 to August 2019, we organized cardiovascular system screenings in three areas of China (the provinces of Liaoning, Shandong and Anhui) for MMA patients who were recruited at local tertiary hospitals (Shengjing Hospital of China Medical University, Jinan Maternity and Child Care Hospital, and Anhui Women and Child Health Care Hospital). During consultation, clinical information was collected, such as the diagnosis (isolated or combined type) according to blood tandem mass spectrometry and urinary gas chromatography, genetic diagnosis, and others.

The control group consisted of age- and gender-matched children who were referred for a pediatric cardiology consultation at XinHua Hospital. The inclusion criteria included minor clinical symptoms (innocent murmur) and normal results on electrocardiography (ECG) and conventional echocardiography. The exclusion criteria included children with a disease or undergoing a treatment that may affect heart function.

### Clinical Assessments and ECG

Height and weight were measured in all subjects. For subjects aged over 3 years old, blood pressure (BP) and heart rate (HR) was measured during rest using an Omron HBP-1,300 professional blood pressure monitor (Omron Healthcare, Guangzhou, CHINA) (19) in the supine position.

Standard 12-lead ECG was performed with the subject in the supine position, and the results were analyzed by a professional medical officer.

### Conventional Echocardiography and STE

Transthoracic echocardiography examinations were performed by a single senior echocardiologist using a CX 50 ultrasound system (Philips Healthcare, Andover, USA). Image acquisition was conducted including M-mode, spectral Doppler flow, tissue Doppler imaging (TDI) (20, 21), and three to five cardiac-cycle loops in apical four-, three-, and two-chamber views for STE analysis (16). S 8-3 and S 5-1 probes (Philips Healthcare, Andover, USA) were used according to the age and weight of the subject.

For each subject, the velocity of tricuspid regurgitation (V_TR_) was quantified if present. The pulmonary arterial systolic pressure (PASP) was calculated by the modified Bernoulli equation, and PAH was diagnosed if PASP >40 mmHg (22).

The left ventricular internal diastolic diameter (LVIDD) was measured using M-mode in the parasternal short-axis view. The LV mass (LVM) was calculated by Devereux's formula (23), and the LVM index (LVMI) (24) was calculated by dividing the LVM by height∧2.7. LV fractional shortening (FS) was calculated as (LVIDD-LVIDS)/LVIDD. The LV ejection fraction (LVEF) was calculated by the cubed method.

All of the following parameters were measured three times in independent cardiac cycles and averaged: the LV early and late diastolic mitral inflow velocity (LV E/ LV A), and peak early diastolic velocity (LV E') at the lateral segment of the mitral annulus; the mean velocity of circumferential fiber shortening corrected for cardiac frequency (mVCFc) and myocardial performance index (MPI); the right ventricular (RV) peak early diastolic velocity (RV E') and early systolic velocity (RV S') measured at the lateral segment of the tricuspid annulus; and the tricuspid annular plane systolic excursion (TAPSE) measured along its longitudinal plane from end-diastole to end-systole.

STE analysis was performed using commercial QLAB version 10.5 software (Philips Healthcare, Andover, USA). The region of interest was anchored as the endocardium in end-diastole, and their longitudinal strain during a heartbeat was detected by the software. The global longitudinal strain (GLS) of the left ventricle was then calculated from the 17-segment model.

### Statistical Analysis

The analysis was performed with EmpowerStats software (version 3.0) and Graphpad Prism (12.0). Continuous variables are presented as the mean ± SD, and categorical variables are presented as frequencies or proportions. To minimize the influence of age, height, weight and body mass index (BMI) were converted to a Z score using the Growth Charts^UK−WHO^ application (version 2.0.1) with references, including the Neonatal and Infant Close Monitoring Growth Chart, the UK WHO 0–4 years' growth chart, and the UK Growth chart (2–18). Comparisons between the two groups were performed using the *t*-test or Kruskal-Wallis rank-sum test for continuous data and chi-squared test for categorical data. Multiple regression analysis was used to identify echocardiography variables affected by the exposure of different types; model I was adjusted for gender, age, and BMI Z score, and model II was adjusted for gender, age, BMI Z score, BP, and HR. A *p* value < 0.05 was considered statistically significant.

## Results

### Population and Baseline Characteristics

A total of 99 patients and 80 control subjects were recruited in this study, with 23 isolated MMA and 76 combined MMA; of them, 84 patients underwent genetic testing and variant identification, and all identified combined MMA patients were the CblC type (Supplementary Figure 1 and Table 1). The basic characteristics were summarized in Table 1. No difference was observed in age, gender, height Z score or BP between three groups. Compared to the controls, the weight Z score (0.4 ± 1.2 vs. −0.3 ± 1.5 vs. −0.3 ± 1.2, *P* = 0.001) and BMI Z score (0.1 ± 1.6 vs. −0.2 ± 1.7 vs. −0.6 ± 1.5, *P* = 0.003) were decreased in both types of MMA patients, which was consistent with their limited developmental state. The heart rate of patients was elevated compared with that of controls (94.4 ± 13.7 vs. 107.6 ± 22.4 vs. 111.7 ± 19.5, *P* < 0.001).

**Table 1:** Baseline characteristics of control, isolated MMA, and MMA/HCY groups.

**Characteristics**	**Control subjects (*N* = 80)**	**Isolated MMA (*N* = 23)**	**MMA/HCY (*N* = 76)**	*P* ^*^
Age (year)	4.1 ± 2.7	6.0 ± 6.6	3.5 ± 2.9	0.226
Male	46	15	38	0.265
Weigh Z score	0.4 ± 1.2	−0.3 ± 1.5	−0.3 ± 1.2	0.001
Height Z score	0.5 ± 1.4	−0.2 ± 1.4	0.2 ± 1.3	0.126
BMI Z score	0.1 ± 1.6	−0.2 ± 1.7	−0.6 ± 1.5	0.003
SBP (mmHg)	97.7 ± 11.2	102.2 ± 18.3	97.5 ± 10.3	0.733
DBP (mmHg)	55.6 ± 10.4	61.6 ± 11.8	58.2 ± 9.8	0.079
HR (bpm)	94.4 ± 13.7	107.6 ± 22.4	111.7 ± 19.5	<0.001

*Data are expressed as the mean ± SD. BMI, body mass index; SBP, systolic blood pressure; DBP, diastolic blood pressure; HR, heart rate*.

* *According to t-test or Kruskal-Wallis test for continuous data and chi-squared test for categorical data, P <0.05 is considered significantly different between groups*.

### Electrocardiography

On ECG, 94 patients have been examined and detailed parameters were listed in Table 2, which were within normal range in both groups. Although no clinically significant changes were found such as premature ventricular contraction, we have noted 11 cases of sinus tachycardia, 1 prolonged QTc interval, 1 I-degree atrioventricular block, 5 cases of left axis deviation, and 14 cases of T wave change such as higher T waves or flat T wave tops with notch.

**Table 2:** Echocardiographic parameters of isolated MMA and MMA/HCY groups.

**Parameters**	**Isolated MMA (*N* = 21)**	**MMA/HCY (*N* = 73)**
P-R interval (ms)	124.4 ± 20.1	110.6 ± 18.1
QRS (ms)	77.9 ± 9.5	73.1 ± 8.5
QTc interval (ms)	312.0 ± 37.6	298.6 ± 31.9
QRS axis (degree)	63.7 ± 24.1	58.8 ± 39.7

*Data are expressed as the mean ± SD*.

### Echocardiographic Variables

Table 3 demonstrated the echocardiographic variables in three groups. Among indexes reflecting left ventricular size, the LVIDD and LVIDS were much smaller in combined type compared to control group and isolated type (LVIDD: 34.7 ± 4.8 vs. 35.1 ± 8.4 vs. 30.9 ± 6.0, *P* < 0.001; LVIDS: 22.2 ± 3.2 vs. 22.2 ± 6.0 vs. 19.8 ± 4.1, *P* < 0.001), indicating a hypogenetic heart in this group, while the LVMI showed no difference between them. The LVEF was within normal range in three groups, although MMA patients had higher LVEF than controls (69.0 ± 9.2 vs. 74.9 ± 4.4 vs. 73.6 ± 4.2, *P* < 0.001). The other two traditional systolic function indexes, LVFS and mVCFc, were not significantly different among three groups, while GLS, a new sensitive index, was reduced in MMA patients (22.7 ± 1.8 vs. 20.3 ± 2.6 vs. 21.3 ± 1.9, *P* < 0.001). This indicator suggested impaired myocardial systolic function. The ratio of LV E/A, an index reflecting LV diastolic function, showed significant reduction in patients compared with control subjects (1.6 ± 0.3 vs. 1.5 ± 0.3 vs. 1.4 ± 0.2, *P* = 0.002), which was consistent with the LV E' velocity (16.0 ± 3.1 vs. 14.5 ± 2.5 vs. 13.1 ± 3.3, *P* < 0.001).

**Table 3:** Echocardiographic variables of control, isolated MMA, and MMA/HCY groups.

	**Echocardiographic variables**	**Control subjects (*N* = 80)**	**Isolated MMA (*N* = 23)**	**MMA/HCY (*N* = 76)**	*P* ^*^
LV	LVIDD (mm)	34.7 ± 4.8	35.1 ± 8.4	30.9 ± 6.0	<0.001
	LVIDS (mm)	22.2 ± 3.2	22.2 ± 6.0	19.8 ± 4.1	<0.001
	IVS (mm)	6.4 ± 1.1	6.7 ± 1.8	6.2 ± 1.3	0.269
	IVS/D	1.5 ± 0.2	1.5 ± 0.2	1.5 ± 0.3	0.369
	EF (%)	69.0 ± 9.2	74.9 ± 4.4	73.6 ± 4.2	<0.001
	FS (%)	35.9 ± 4.6	37.1 ± 3.9	35.9 ± 3.6	0.733
	LVM (g)	34.3 ± 13.5	41.7 ± 36.0	27.2 ± 17.9	<0.001
	LVMI (g/m^2.7^)	31.7 ± 8.9	31.9 ± 10.4	29.5 ± 9.6	0.100
	E/A ratio	1.6 ± 0.3	1.5 ± 0.3	1.4 ± 0.2	0.002
	E' (cm/s)	16.0 ± 3.1	14.5 ± 2.5	13.1 ± 3.3	<0.001
	mVCFc (sec^−1^)	1.1 ± 0.1	1.1 ± 0.2	1.1 ± 0.1	0.841
	MPI	0.2 ± 0.1	0.3 ± 0.1	0.2 ± 0.1	0.360
	GLS (%)	22.7 ± 1.8	20.3 ± 2.6	21.3 ± 1.9	<0.001
RV	E'	14.8 ± 3.0	15.9 ± 4.4	14.5 ± 3.5	0.509
	S'	12.7 ± 2.2	14.0 ± 2.5	12.9 ± 1.8	0.033
	TAPSE	19.0 ± 3.2	19.6 ± 4.0	17.2 ± 3.2	<0.001

*Data are expressed as the mean ± SD. LV, left ventricle; RV, right ventricle; LVIDD, left ventricular internal diastolic diameter; LVIDS, left ventricular internal systolic diameter; IVS, interventricular systolic septum; IVS/D, ratio of interventricular systolic and diastolic septum; EF, ejection fractio; FS, fractional shortening; LVM, left ventricular mass; LVMI, left ventricular mass index; E/A, ratio of early and late diastolic mitral inflow velocity; E', peak early diastolic velocity; S', peak early systolic velocity; mVCFc, mean velocity of circumferential fiber shortening; MPI, myocardial performance index; GLS, global longitudinal strain; TAPSE, tricuspid annular plane systolic excursion*.

* *According to t-test or Kruskal-Wallis test for continuous data, P < 0.05 is considered significantly different between groups*.

For the right ventricle, the TAPSE, an index of RV systolic function, showed a significant reduction in combined type compared to the others (19.0 ± 3.2 vs. 19.6 ± 4.0 vs 17.2 ± 3.2, *P* < 0.001), while another systolic index RV S' showed a different tendency (12.7 ± 2.2 vs. 14.0 ± 2.5 vs. 12.9 ± 1.8, *P* = 0.033). As for diastolic function, there was no significant difference in RV E' among three groups.

### Multiple Regression Analysis

As results showed above, the changing tendency of cardiac function among three groups can be quite conflicting according to different echocardiographic variables. Thus, we have done multiple regression analysis to rule out other factors such as age and gender to clarify the influence of exposure of MMA subtypes on cardiac function.

**Figure 2:**
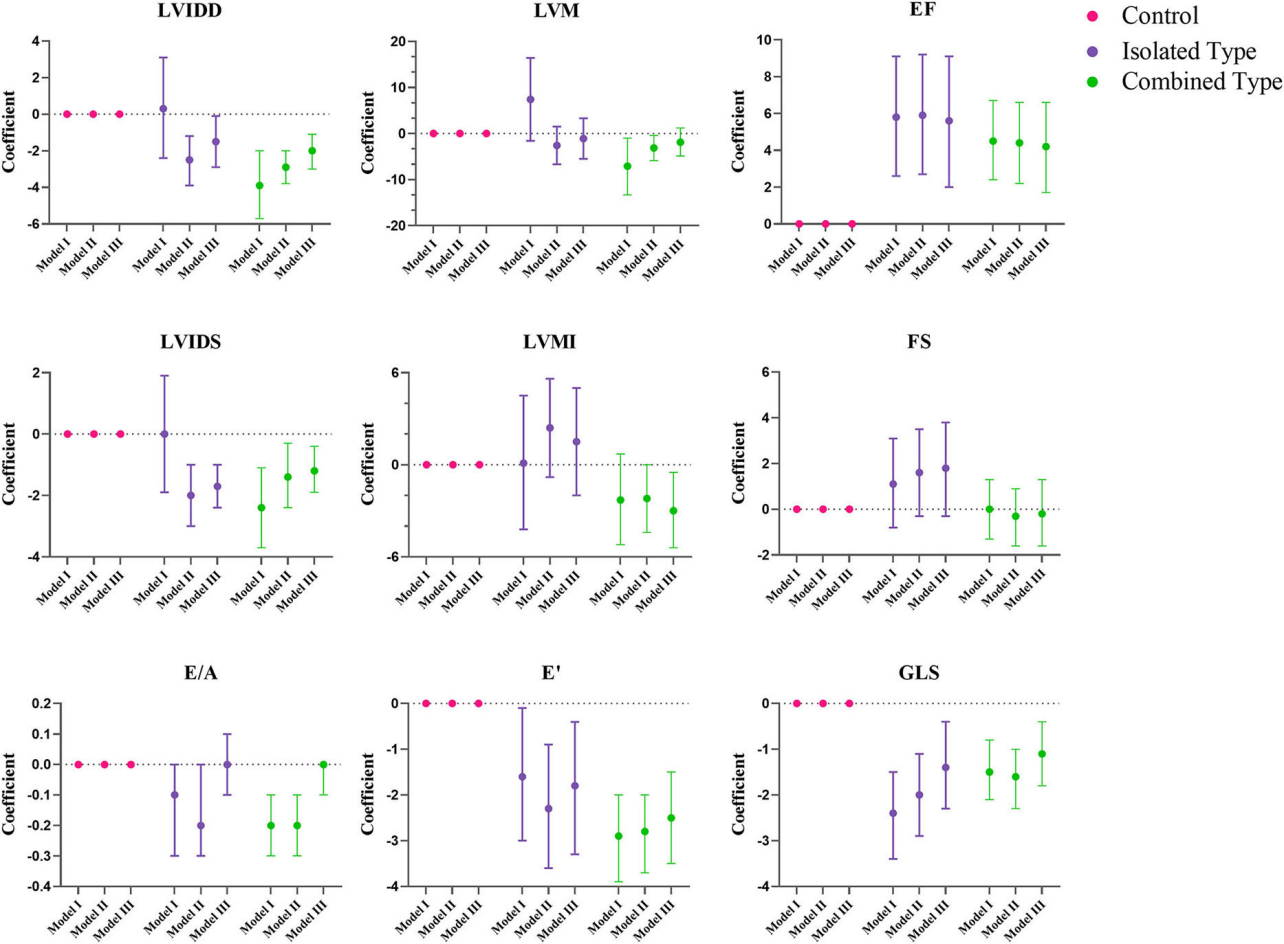
Multiple regression analysis of left ventricular echocardiographic variables between control, isolated MMA and combined MMA patients. Multiple regression analysis was used with control group as reference. Model I was not adjusted, model II was adjusted for age, gender and BMI Z score, and model III was adjusted for age, gender, BMI Z score, blood pressure and heart rate. LVIDD, left ventricular internal diastolic diameter; LVIDS, left ventricular internal systolic diameter; EF, ejection fraction; FS, fractional shortening; LVM, left ventricular mass; LVMI, left ventricular mass index; E/A, ratio of early and late diastolic mitral inflow velocity; E', peak early diastolic velocity; GLS, global longitudinal strain.

**Figure 3:**
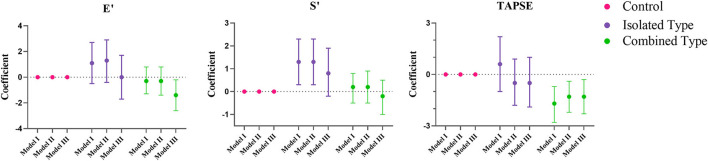
Multiple regression analysis of right ventricular echocardiographic variables between control, isolated MMA and combined MMA patients. Multiple regression analysis was used with control group as reference. Model I was not adjusted, model II was adjusted for age, gender and BMI Z score, and model III was adjusted for age, gender, BMI Z score, blood pressure and heart rate. E', peak early diastolic velocity; S', peak early systolic velocity; TAPSE, tricuspid annular plane systolic excursion.

Figures 2, 3 showed the regression coefficients and 95% confidence intervals, detailed values were in Supplementary Table 2. According to model III, LVIDD, and LVIDS both diminished due to exposure of isolated or combined MMA (LVIDD: isolated [*R* = −1.5, 95%CI (−2.9, −0.1), *P* = 0.036] combined [*R* = −2.0, 95%CI (−3.0, −1.1), *P* < 0.001]; LVIDS: isolated [*R* = −1.7, 95%CI (−2.4, −1.0), *P* < 0.001] combined [*R* = −1.2, 95%CI (−1.9, −0.4), *P* = 0.002)], while LVMI showed significant reduction only in combined type [*R* = −3.0, 95%CI (−5.4, −0.5), *P* = 0.017]. LVEF still remained elevated [isolated: *R* = 5.6, 95%CI (2.0, 9.1), *P* = 0.002; combined: *R* = 4.2, 95%CI (1.7, 6.6), *P* = 0.001], and GLS reduced [isolated: *R* = −1.4, 95%CI (−2.3, −0.4), *P* = 0.007; combined: *R* = −1.1, 95%CI (-1.8, −0.4), *P* = 0.001] in both MMA groups. Although the ratio E/A showed no more difference, the LV E' velocity still showed significant reduction in both MMA groups [isolated: *R* = −1.8, 95%CI (−3.3, −0.4), *P* = 0.016; combined: *R* = −2.5, 95%CI (−3.5, −1.5), *P* < 0.001]. As for right ventricle, only combined type had significant reduction in TAPSE [*R* = −1.3, 95%CI (−2.3, −0.3); *P* = 0.013], and RV E' [*R* = −1.4, 95%CI (−2.6, −0.2); *P* = 0.021].

### Clinical Cardiovascular Involvement

During our echocardiography examination, 8 combined MMA patients with cardiovascular involvement were noted, accounting for 10.5% of this population, and their detailed information was listed in Table 4. There were eight cases of CHD [including two ventricular septal defects (VSD), three atrial septal defects (ASD), one patent ductus arteriosus (PDA), and two coronary artery-pulmonary artery fistulas (CA-PAF)], one case of pulmonary hypertension, and one case of hypertrophic cardiomyopathy.

**Table 4:** Clinical information of combined MMA patients with cardiovascular involvement.

**Patient**	**1**	**2**	**3**	**4**	**5**	**6**	**7**	**8**
Gender	Female	Female	Male	Male	Female	Male	Male	Male
Age at onset	1 month	1 month	NA	NA	Birth	3 months	6 years	4 years
Age at diagnosis	2 months	4 months	15 days	20 days	1 month	2 years and 3 months	6 years	4 years
*MMACHC* variant	c.567dupT/ c.567dupT	c.609G> A/c.658_660delAAG	NA	c.609G>A/c.609G>A	c.609G>A/c.616C>T	c.609G>A/c.80A>G	c.80A>G/ c.217C>T	c.609G>A/c.80A>G
Cardiovascular involvement	Muscular VSD/CA-PAF	ASD/PDA	ASD/VSD/LV enlargement	CA-PAF	LV hypertrophy	PAH/heart failure	Systemic hypertension/heart failure	PAH
Other complications	/	/	/	/	/	CKD I/anemia	Haematuria/ proteinuria/ anemia	AHS/hypothyroidism

*ASD, atrial septal defect; VSD, ventricular septal defect; PDA, patent ductus arteriosus; CA-PAF, coronary artery-pulmonary artery fistula; AHS, alveolar haemorrhagic syndrome; CKD, chronic kidney disease; PAH, pulmonary arterial hypertension; NA, not available*.

Patient 1 had a muscular VSD with a diameter of 1.8 mm and a CA-PAF. Patient 2 had a ASD and a PDA. Patient 3 had two ASDs (2.5 mm and 3.5 mm, respectively) and a VSD, which were responsible for the enlargement of the LV and required an interventional operation for defect closure. Patient 4 had a CA-PAF, and his left coronary artery was slightly enlarged, with a diameter of 3.13 mm. Patient 5 had a relatively thick interventricular septum for her age as the IVS during systolic phase was 7.16 mm and the Z score was +1.52.

Patient 6 presented at 3 months of age with jaundice and failure to thrive and had shown progressive cyanosis since 1.5 years of age. He was admitted at 2 years and 3 months of age due to oedema and worsening cyanosis. The CblC type was diagnosed genetically with variants (c.609G>A/c.80A>G). Echocardiography revealed PAH (WHO FC IV) with a PASP of 96 mmHg and a significantly dilated right ventricle. With treatment with PAH-targeted drugs and hydroxocobalamin, this patient gradually recovered and showed no recurrence of PAH during follow-up.

Patient 7 was admitted at 6 years of age due to systemic hypertension, haematuria, proteinuria, and anemia to a local hospital, and echocardiography revealed LV hypertrophy, and enlargement, impairment of global LV systolic (EF, 51; FS, 26%), and diastolic function with no signs of PAH, which was probably secondary to the systemic hypertension and anemia. This patient was then transferred to a higher level hospital and diagnosed with the CblC type harboring heterozygous variants (c.80A>G/c.217c>T). Renal biopsy revealed endocapillary proliferative glomerulonephritis. After treatment with antihypertensive drugs, nephroprotective agents and hydroxocobalamin, control of this patient's blood pressure was achieved, with improved cardiac function and negative conversion of haematuria and proteinuria.

Patient 8 first showed narcolepsy, vomiting, jaundice, anemia and dyspnoea at 4 years of age and was then diagnosed with the CblC type harboring variants (c.609G>A/c.80A>G). 1 month later, this child was transferred to the ICU due to the sudden onset of respiratory failure. He presented with hypovolemic shock and decompensated metabolic acidosis, and fibreoptic bronchoscopy with bronchoalveolar lavage indicated alveolar haemorrhagic syndrome (AHS). Echocardiography revealed moderate PAH with a PASP of 62 mmHg and LV enlargement with impaired mobility. In addition, a venous thrombus was detected in the left superficial jugular vein. With aggressive symptomatic treatment and hydroxocobalamin, this patient was rescued.

As mentioned above, patients 6–8 shared a common *MMACHC* variant, c.80A>G. In addition, only patient 6 had mild PAH, while the others showed no signs of PAH at the time of echocardiography.

## Discussion

### Myocardial Dysfunction

According to our study, the heart was smaller in both MMA subtypes, as the LVIDD and LVMI were lower in the patients than the control subjects, this may play a role in their increased heart rate as higher frequency was required to fulfill their physiological need for circulating a sufficient blood volume.

In terms of systolic function, the LV GLS showed a reduction of 1.4 and 1.1% in isolated MMA and combined MMA group, respectively, indicating impaired systolic function, although the EF was increased in all MMA patients. This inconsistency may be due to the cubed method that presumes the heart as a cube which is not an accurate model. In addition, we have analyzed the relationship of GLS and age in different groups (Supplementary Figure 2) by linear regression model. Both isolated and combined MMA groups had a significant lower intercept compared to control group, meanwhile the combined MMA group showed a steeper slope suggesting that this group suffered a more severe adverse effect along with time. And the results of TAPSE suggested that RV systolic function was impaired only in combined MMA patients. As for diastolic function, in combined MMA patients, our study showed a lower E' velocity in both ventricles, while isolated MMA group only exhibited reduced LV E'. Thus we summarized that isolated MMA and combined MMA group showed different pattern of cardiac function impairment, in which the former only affected the left ventricle while the latter affected both ventricles.

Although the pathophysiology of this disease remains unclear, there are several explanatory hypotheses, including direct toxicity of excess metabolites (methylmalonic acid and homocysteine), enhanced oxidative stress and mitochondrial disorders (1, 25–27). For isolated MMA, the pathogenic mechanism may be closer to that of propionic acidaemia, which is mainly related to the disorder of the tricarboxylic acid cycle and the damage of the respiratory chain. Due to direct cytotoxic effects of accumulated propionic acid and methylmalonyl, the level of reactive oxygen species increases dramatically with reduced activity of antioxidant enzymes, causing other side-effects such as lipid peroxidation, protein carbonylation, and oxidation of mitochondrial DNA. In addition, extra-accumulation of mitochondrial permeability transition pore increases the non-selective permeability of the mitochondrial membrane, leading to the loss of reduced coenzymes I and II, calcium ions, which reduces membrane potential, and causes mitochondrial oedema. These processes severely damage the function of mitochondria, and then promote the synthesis of reactive oxygen species, thus forming a vicious circle. On the other hand, the accumulation of methylmalonic acid and propionate competitively consumes CoA to synthesize methylmalonyl-CoA and propionyl-CoA, while the heart tissue lacks the corresponding carnitine acyltransferase and cannot release CoA by displacement reaction. Thus, the tricarboxylic acid cycle is inhibited, resulting in a cardiac metabolism transition from fatty acid oxidation to sugar catabolism, which is similar in patients with heart failure (28, 29).

For combined MMA patients, extra mechanisms relating homocysteine may be involved. Hyperhomocysteinaemia can induce endothelial-myocyte uncoupling by matrix metalloproteinase activation and subsequent interstitial fibrosis accumulation, and this uncoupling leads to impaired diastolic relaxation (30, 31). In addition, beta2-adrenergic receptor was found to be down-regulated due to homocysteine, which contributed to the impaired contractile function of cardiomyocytes in diabetic cardiomyopathy (32).

### Higher Prevalence of CHD

In our study, combined MMA patients showed a much higher incidence of CHD of ~5.3% compared to that of 8.98 per 1,000 live births in the general population in China (33).

In CblC patients, because of insufficient MeCbl, the remethylation of homocysteine (Hcy) to methionine (Met) catalyzed by methionine synthase is greatly impaired, causing disturbance or disruption of the Met-Hcy-SAM pathway. S-adenosylmethionine (SAM) is an important methyl group donor and participates in various methylation reactions, including DNA and histone methylation (34, 35). A growing body of research has shown a strong connection between DNA or histone methylation disorders and the occurrence of CHD. A case-control study conducted by Sylvia et al. (36) pointed out that Down syndrome and CHD may be associated with a global hypomethylation status, as they showed a higher S-adenosylhomocysteine (SAH) level and lower SAM: SAH ratios. Through the analysis of genome-wide DNA methylation data from myocardial biopsies in CHD patients, Marcel et al. (37) found that the aberrant methylation of promoter CpG islands and methylation alterations could result in differences in DNA splicing and contribute to the occurrence of CHD. Apart from DNA methylation, histone methylation modification, as an important epigenetic regulatory component, has been verified to be involved in the development of heart and blood vessels. Variants and deficiencies in histone methylation-modifying enzymes have resulted in various cardiac abnormalities in different species (38). Moreover, the *MMACHC* gene itself has shown tissue-specific expression in the developing heart in mouse embryos, indicating its involvement in cardiac development (39). An *MMACHC* proteomic analysis suggested that CblC variants led to broad metabolic dysfunction, including dysregulation of the cytoskeleton and cell signaling. Pathway analysis demonstrated a strong association with cardiovascular disease, especially cardiomyopathy, due to excessive collagen production (40).

### Potential Thrombus-Related Diseases

Another form of cardiovascular involvement is acute heart failure, which is more critical and life-threatening. To date, the associated variants have shown strong heterogeneity among races. In European countries, c.271dupA and c.276G>T were the two leading variants detected in CblC patients with isolated PAH or a combination of PAH and atypical haemolytic uraemic syndrome (aHUS) (6, 10, 41, 42), while in China, c.80A>G was the leading variant detected (8, 9, 43, 44). In a study including 15 MMA/HCY patients with PAH, genetic diagnosis was performed in ten patients, and all of them carried the *MMACHC* c.80A>G variant (43). Our group also collected twelve CblC type patients with PAH, and only one patient did not have the c.80A>G variant (data unpublished). The strong connection of this variant with PAH should be further investigated.

The mechanism was suspected to be hyperhomocysteinaemia-related thrombotic microangiopathy (TMA), as thrombi were detected in pulmonary vessels and on renal biopsy, and homocysteine was recognized as a risk factor for arteriosclerosis and thrombosis in adults. However, there was controversy as thrombosis was not found in all patients with PAH, and some patients presented with interstitial lung disease or pulmonary vessel abnormalities (9, 43, 44). In our study, patient 8 was diagnosed with AHS and showed no indications of pulmonary embolism. These diverse clinical manifestations suggest the involvement of different mechanisms, including thrombosis, endothelial damage and vascular dysplasia. In addition, pulmonary hypertension was also reported in isolated MMA patients (45, 46), although the incidence was lower; thus, there may be a synergistic mechanism of involving methylmalonic acid and homocysteine in CblC patients leading to this clinical manifestation.

## Conclusion

Isolated and combined MMA groups showed different pattern of cardiac function impairment, in which the former only affected the left ventricle while the latter affected both ventricles, and affected ventricle exhibited both systolic and diastolic function impairment. In addition, there was a relatively high incidence of CHD in combined MMA group, and we recommended that combined MMA patients should undergo routine cardiovascular examinations. For patients carrying the *MMACHC* c.80A>G variant, extra attention should be paid to signs of PAH.

## Data Availability Statement

The raw data supporting the conclusions of this article will be made available by the authors, without undue reservation.

## Ethics Statement

The studies involving human participants were reviewed and approved by Ethics Committee of Xinhua Hospital Affiliated to Shanghai Jiaotong University School of Medicine. Written informed consent to participate in this study was provided by the participants' legal guardian/next of kin.

## Author Contributions

YW, KS, and LH contributed equally to the study and conceived and designed the study. YL and LY prepared an analytical plan, analyzed data, and drafted the initial manuscript. RS was involved in the clinical data collection. SH and BZ were involved in the electrocardiography. YW performed the echocardiography examinations. All authors have reviewed and revised the manuscript, approved the final manuscript as submitted, and agreed to be accountable for all aspects of the work.

## Funding

This work was supported by the Program of Shanghai Municipal Health Commission (No. 202040160) and the Program of Shanghai Municipal Commission of Health and Family Planning (No. 201840347).

## Conflict of Interest

The authors declare that the research was conducted in the absence of any commercial or financial relationships that could be construed as a potential conflict of interest.

## Publisher's Note

All claims expressed in this article are solely those of the authors and do not necessarily represent those of their affiliated organizations, or those of the publisher, the editors and the reviewers. Any product that may be evaluated in this article, or claim that may be made by its manufacturer, is not guaranteed or endorsed by the publisher.

## Supplementary Material

Supplementary Figure 1 Enrolment of MMA patients. 99 MMA patients enrolled, including 23 isolated MMA patients and 76 combined MMA patients. Isolated MMA patients consisted of 18 patients caused by methylmalonyl-CoA mutase (MUT) variant (OMIM# 251000), 1 MMAA variant (OMIM# 251100), and 1 MMAB variant (OMIM# 251110). 64 combined MMA patients had identified *MMACHC* variant (OMIM #277400).

Supplementary Figure 2 Effect of age on the GLS in control, isolated MMA and combined MMA groups.
