# Supplementary figures and images for: Different Pattern of Cardiovascular Impairment in Methylmalonic Acidaemia Subtypes
Source: Front Pediatr. 2022 Feb 23;10:810495. doi: 10.3389/fped.2022.810495 (PMC8904414; doi:10.3389/fped.2022.810495)

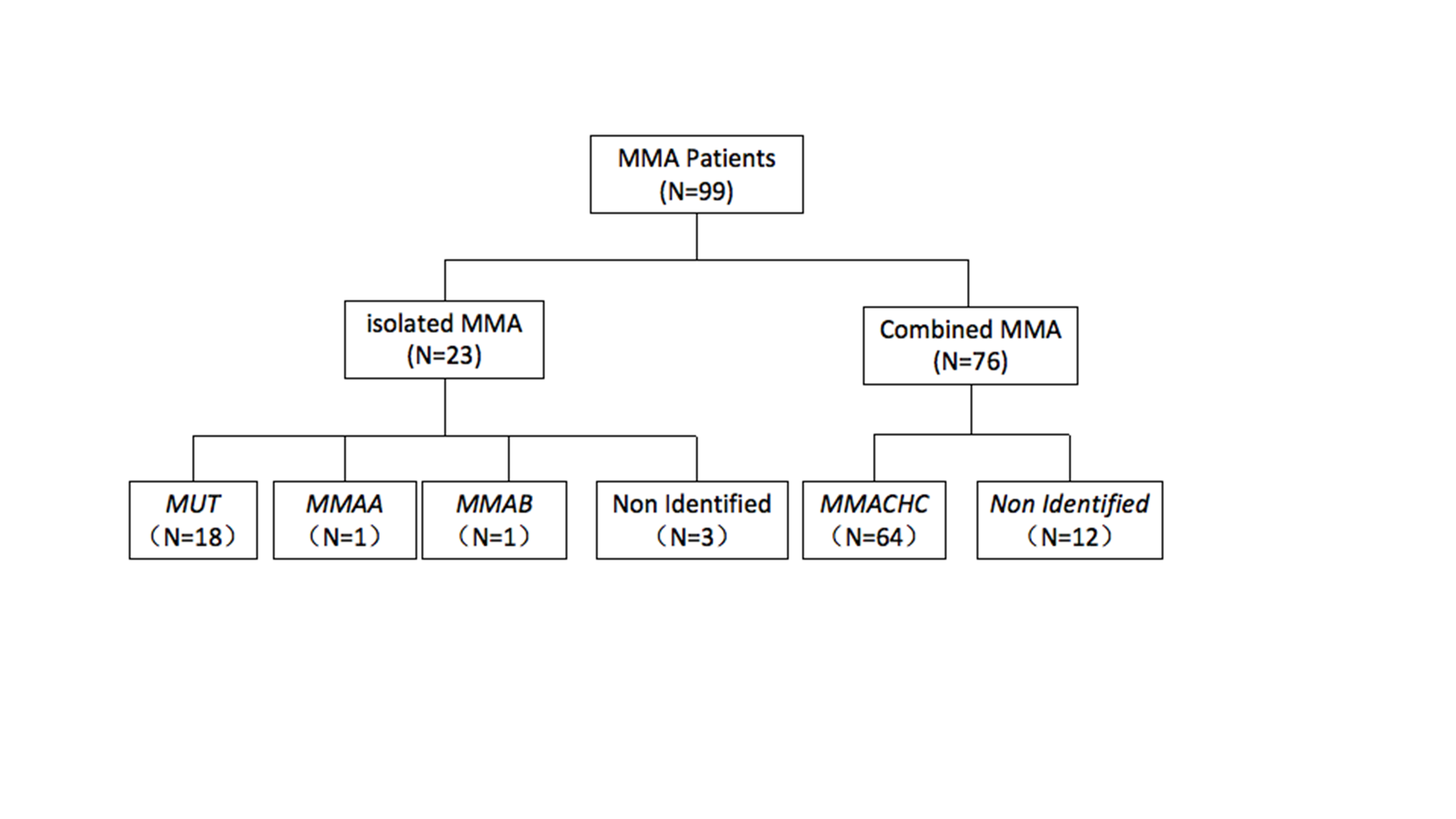

Supplement: Supplementary Figure 1 — Enrolment of MMA patients. 99 MMA patients enrolled, including 23 isolated MMA patients and 76 combined MMA patients. Isolated MMA patients consisted of 18 patients caused by methylmalonyl-CoA mutase (MUT) variant (OMIM# 251000), 1 MMAA variant (OMIM# 251100), and 1 MMAB variant (OMIM# 251110). 64 combined MMA patients had identified MMACHC variant (OMIM #277400). [file Image_1.TIF]

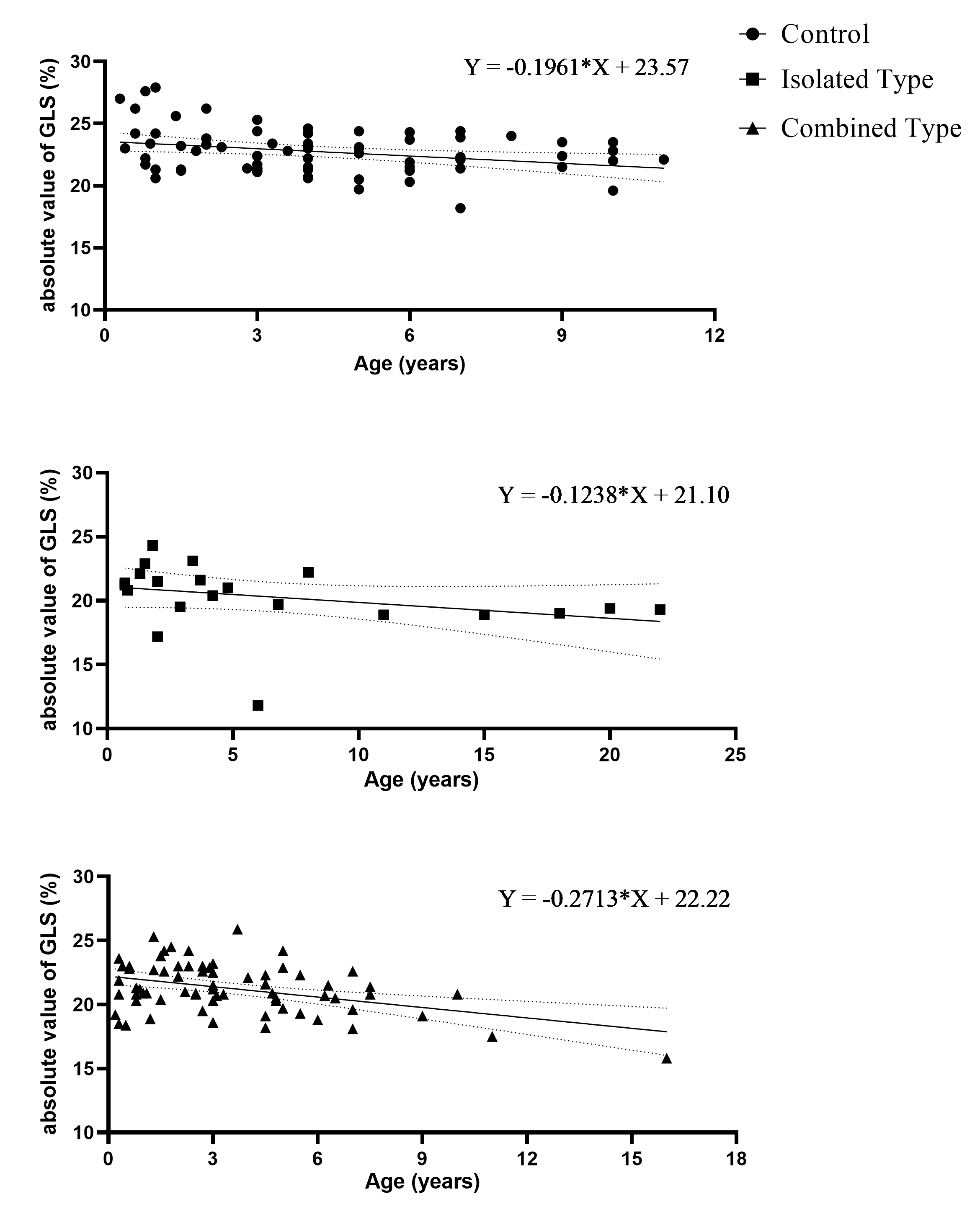

Supplement: Supplementary Figure 2 — Effect of age on the GLS in control, isolated MMA and combined MMA groups. [file Image_2.TIF]
