# Supplementary material for: Different Pattern of Cardiovascular Impairment in Methylmalonic Acidaemia Subtypes
Source: Front Pediatr. 2022 Feb 23;10:810495. doi: 10.3389/fped.2022.810495 (PMC8904414; doi:10.3389/fped.2022.810495)
Supplement: Supplementary file 3 [file Data_Sheet_1.docx]

Supplementary table 1. Detection of gene variants in combined MMA patients

| Exon | Variants | Amino acid change | Type | Clinical significance | Frequency | Percentage |
| --- | --- | --- | --- | --- | --- | --- |
| E4 | c.609G>A | p.W203X | Nonsense | Pathogenic | 52 | 40.6% |
| E4 | c.658_660delAAG | p.K220del | Deletion | Pathogenic | 15 | 10.8% |
| E4 | c.656_658delAGA | p.219_220del | Deletion | Pathogenic | 14 | 10.1% |
| E1 | c.80A>G | p.Q27R | Missense | Pathogenic | 8 | 6.2% |
| E3 | c.394C>T | p.R132X | Nonsense | Pathogenic | 7 | 5.4% |
| E4 | c.567dupT | p.I190Yfs*13 | Frame shift | Pathogenic | 6 | 4.6% |
| E2 | c.217C>T | p.R73X | Nonsense | Pathogenic | 5 | 3.9% |
| E4 | c.445_446delTG | p.C149Hfs*32 | Frame shift | Pathogenic | 4 | 3.0% |
| E4 | c.481C>T | p.R161X | Nonsense | Pathogenic | 2 | 1.6% |
| E3 | c.331C>T | p.R111X | Nonsense | Pathogenic | 2 | 1.6% |
| E2 | c.228_231delTGAC | p.D77Qfs*22 | Frame shift | Pathogenic | 1 | 0.8% |
| E3 | c.315C>G | p.Y105X | Nonsense | Pathogenic | 1 | 0.8% |
| E4 | c.445_446insA | p.C149X | Nonsense | Pathogenic | 1 | 0.8% |
| E4 | c.484G>A | p.G162R | Missense | Pathogenic | 1 | 0.8% |
| E4 | c.566_567insT | p.R189Rfs*14 | Frame shift | Pathogenic | 1 | 0.8% |
| E4 | c.616C>T | p.R206W | Missense | Pathogenic | 1 | 0.8% |
| E4 | c.624_625del | p.A208fs | Frame shift | Likely pathogenic | 1 | 0.8% |
| E4 | c.626_627delTG | p.V209Dfs*35 | Frame shift | Likely pathogenic | 1 | 0.8% |
| E4 | c.626dupT | p.T210Dfs*35 | Frame shift | Pathogenic | 1 | 0.8% |
| E4 | c.634delC | p.Q212Rfs*79 | Frame shift | Pathogenic | 1 | 0.8% |
| E1 | E1 deletion | / | Deletion | Pathogenic | 1 | 0.8% |
| / | Not identified | / | / | / | 2 | 1.6% |

Supplementary table 2. Multiple regression analysis of echocardiographic variables between control, isolated MMA and combined MMA patients

| Echocardiographic variables | | Unadjusted Model | | Adjusted Model I | | Adjusted Model II | |
| --- | --- | --- | --- | --- | --- | --- | --- |
|  |  | Isolated MMA | MMA/HCY | Isolated MMA | MMA/HCY | Isolated MMA | MMA/HCY |
| LV | LVIDD | 0.3 (-2.4, 3.1)；0.813 | -3.9 (-5.7, -2.0)；<0.001 | -2.5 (-3.9, -1.2)；<0.001 | -2.9 (-3.8, -2.0)；<0.001 | -1.5 (-2.9, -0.1)；0.036 | -2.0 (-3.0, -1.1)；<0.001 |
|  | LVIDS | -0.0 (-1.9, 1.9)；0.970 | -2.4 (-3.7, -1.1)；<0.001 | -2.0 (-3.0, -1.0)；<0.001 | -1.4 (-2.4, -0.3)；0.010 | -1.7 (-2.4, -1.0)；<0.001 | -1.2 (-1.9, -0.4)；0.002 |
|  | IVS | 0.2 (-0.4, 0.8)；0.467 | -0.2 (-0.6, 0.2)；0.334 | -0.4 (-0.8, 0.0)；0.085 | 0.0 (-0.2, 0.3)；0.798 | -0.2 (-0.6, 0.3)；0.421 | 0.2 (-0.1, 0.5)；0.237 |
|  | IVS/D | -0.1 (-0.2, 0.1)；0.283 | -0.0 (-0.1, 0.0)；0.365 | -0.1 (-0.2, 0.1)；0.281 | -0.0 (-0.1, 0.0)；0.356 | -0.1 (-0.2, 0.1)；0.435 | -0.0 (-0.1, 0.1)；0.570 |
|  | EF | 5.8 (2.6, 9.1)；<0.001 | 4.5 (2.4, 6.7)；<0.001 | 5.9 (2.7, 9.2)；<0.001 | 4.4 (2.2, 6.6)；<0.001 | 5.6 (2.0, 9.1)；0.002 | 4.2 (1.7, 6.6)；0.001 |
|  | FS | 1.1 (-0.8, 3.1)；0.239 | -0.0 (-1.3, 1.3)；0.994 | 1.6 (-0.3, 3.5)；0.104 | -0.3 (-1.6, 0.9)；0.595 | 1.8 (-0.3, 3.8)；0.088 | -0.2 (-1.6, 1.3)；0.821 |
|  | LVM | 7.4 (-1.6, 16.4)；0.109 | -7.1 (-13.3, -1.0)；0.023 | -2.6 (-6.7, 1.5)；0.213 | -3.1 (-5.9, -0.4)；0.027 | -1.1 (-5.5, 3.3)；0.630 | -1.9 (-4.9, 1.2)；0.235 |
|  | LVMI | 0.1 (-4.2, 4.5)；0.956 | -2.3 (-5.2, 0.7)；0.130 | 2.4 (-0.8, 5.6)；0.146 | -2.2 (-4.4, -0.0)；0.047 | 1.5 (-2.0, 5.0)；0.407 | -3.0 (-5.4, -0.5)；0.017 |
|  | E/A | -0.1 (-0.3, 0.0)；0.068 | -0.2 (-0.3, -0.1)；<0.001 | -0.2 (-0.3, -0.0)；0.016 | -0.2 (-0.3, -0.1)；<0.001 | 0.0 (-0.1, 0.1)；0.899 | -0.0 (-0.1, 0.0)；0.366 |
|  | E' | -1.6 (-3.0, -0.1)；0.038 | -2.9 (-3.9, -2.0)；<0.001 | -2.3 (-3.6, -0.9)；0.001 | -2.8 (-3.7, -2.0)；<0.001 | -1.8 (-3.3, -0.4)；0.016 | -2.5 (-3.5, -1.5)；<0.001 |
|  | mVCFc | 0.0 (-0.0, 0.1)；0.510 | -0.0 (-0.1, 0.0)；0.398 | 0.0 (-0.0, 0.1)；0.324 | -0.0 (-0.1, 0.0)；0.221 | 0.0 (-0.0, 0.1)；0.435 | -0.0 (-0.1, 0.0)；0.216 |
|  | MPI | 0.0 (-0.0, 0.1)；0.288 | -0.0 (-0.0, 0.0)；0.386 | 0.0 (-0.0, 0.1)；0.381 | -0.0 (-0.0, 0.0)；0.389 | 0.0 (-0.0, 0.1)；0.410 | -0.0 (-0.0, 0.0)；0.458 |
|  | GLS | -2.4 (-3.4, -1.5)；<0.001 | -1.5 (-2.1, -0.8)；<0.001 | -2.0 (-2.9, -1.1)；<0.001 | -1.6 (-2.3, -1.0)；<0.001 | -1.4 (-2.3, -0.4)；0.007 | -1.1 (-1.8, -0.4)；0.001 |
| RV | E' | 1.1 (-0.5, 2.7)；0.180 | -0.3 (-1.3, 0.8)；0.637 | 1.3 (-0.4, 2.9)；0.137 | -0.3 (-1.4, 0.8)；0.545 | -0.0 (-1.7, 1.7)；0.998 | -1.4 (-2.6, -0.2)；0.021 |
|  | S' | 1.3 (0.3, 2.3)；0.009 | 0.2 (-0.5, 0.8)；0.576 | 1.3 (0.3, 2.3)；0.011 | 0.2 (-0.5, 0.9)；0.558 | 0.8 (-0.2, 1.9)；0.136 | -0.2 (-1.0, 0.5)；0.543 |
|  | TAPSE | 0.6 (-1.0, 2.2)；0.448 | -1.7 (-2.8, -0.7)；0.001 | -0.5 (-1.8, 0.9)；0.475 | -1.3 (-2.2, -0.4)；0.004 | -0.5 (-1.9, 1.0)；0.541 | -1.3 (-2.3, -0.3)；0.013 |

Data are expressed as (Coefficient (95% CI); P), P <0.05 is considered significantly different between groups.

LV: left ventricle; RV: right ventricle; LVIDD: left ventricular internal diastolic diameter; LVIDS：left ventricular internal systolic diameter；IVS：interventricular systolic septum；IVS/D：ratio of interventricular systolic and diastolic septum；EF：ejection fractio；FS：fractional shortening；LVM：left ventricular mass；LVMI：left ventricular mass index；E/A：ratio of early and late diastolic mitral inflow velocity；E'：peak early diastolic velocity；S'：peak early systolic velocity; mVCFc: mean velocity of circumferential fiber shortening; MPI: myocardial performance index；GLS：global longitudinal strain；TAPSE：tricuspid annular plane systolic excursion.

Multiple regression analysis was used; model I was adjusted for age, gender and BMI Z score, and model II was adjusted for age, gender, BMI Z score, blood pressure and heart rate.
